# Adoption of an Electronic Patient Record Sharing Pilot Project: Cross-Sectional Survey

**DOI:** 10.2196/13761

**Published:** 2020-04-06

**Authors:** Jingxuan Wang, Junjie Huang, Clement Shek Kei Cheung, Wing Nam Wong, Ngai Tseung Cheung, Martin CS Wong

**Affiliations:** 1 The Jockey Club School of Public Health and Primary Care Faculty of Medicine New Territories Hong Kong; 2 Information Technology and Health Informatics Division Hospital Authority Hong Kong China

**Keywords:** health information exchange, shared electronic health record, online platform, public-private partnership

## Abstract

**Background:**

The Public Private Interface–Electronic Patient Record (PPI-ePR) system was implemented as a new electronic platform to facilitate collaboration between the public and private sectors in Hong Kong. However, its barriers to participate and benefits have not been comprehensively assessed.

**Objective:**

This study aimed to evaluate the awareness, acceptance, perceived benefits, and obstacles to participation among private doctors and the general public.

**Methods:**

From December 2012 to January 2013, 2435 telephone interviews were performed by trained interviewers to survey randomly selected patients who were enrolled or not enrolled in the PPI-ePR system. In addition, self-administered surveys were sent by postal mail to 4229 registered doctors in Hong Kong. The questionnaires for both patients and doctors contained questions on subjects’ awareness, acceptance, and perceptions of the PPI-ePR, perceived benefits and obstacles of participating in the program, reasons for not using the system after enrolling, and perceived areas for service improvement of the system.

**Results:**

More than 53.1% (266/501) of enrolled patients believed that the PPI-ePR system would improve health care quality by reducing duplicate tests and treatments, while more than 76.8% (314/409) of enrolled doctors emphasized timely access to patients’ medical records as the biggest benefit of their enrollment. Among nonenrolled patients, unawareness of the project was the most popular obstacle to enrolling in the PPI-ePR system (483/1200, 40.3%). Regarding nonenrolled doctors, the complicated registration process hindered them from participating in the program the most (95/198, 48.0%). Television, newspaper, and magazine advertisements and medical profession newsletters or journals were suggested as the most effective means to encourage participation in the program among surveyed patients (1297/1701, 76.2%) and doctors (428/610, 70.2%), respectively. Lack of clinical indication requiring data extraction from other hospitals was the main reason for low level of PPI-ePR use.

**Conclusions:**

This study comprehensively assessed the popularity, perceived benefits, and hindering factors of enrolling in the PPI-ePR system in Hong Kong. Low levels of awareness, few privacy concerns, and inactive use of the PPI-ePR system were among the key features for patients and physicians. Public promotions, simplified logistics, and a user-friendly online interface were suggested to improve the coverage and effectiveness of health information exchange between private and public health care sectors.

## Introduction

The electronic health record systems (EHRs) and electronic medical records (EMRs) have been widely discussed in Western societies [1-5]. Promotion of these systems is believed to facilitate the communication between doctors and patients, reduce health care costs, enhance medical efficacy, activate patients to join in their holistic care, and support patient self-management of health [6-10]. Assessment of factors affecting participation in and adoption of local electronic systems, however, has scarcely been done, especially in Asian societies [11-15].

The health care system in Hong Kong runs on a dual-track basis encompassing the public and private sectors. While the 44 public hospitals provide approximately 90% of hospital medical service and 29% of outpatient medical service, the private health care sector provides personalized choices and more accessible services to those who are willing and can afford to pay [16]. High mobility between hospitals was observed among both patients and doctors [17]. The Hospital Authority (HA) of Hong Kong, the statutory administrative body that manages all public hospitals and health institutes in Hong Kong, determined that the overburdened public hospitals had long waiting times and ambiguous rules and procedures on patient referral between private and public sectors [16]. Paper-based data exchange among hospitals and clinics required a cumbersome process and was subject to safety and quality issues in many cases. Australia, Malaysia, and many Western countries have developed hospital information management and exchange systems to facilitate cooperation between private and public health care sectors [18]. In Hong Kong, the Public-Private Interface-Electronic Patient Record (PPI-ePR) program was thus implemented in 2006 to promote a public-private partnership and enhance timely, large-scale, secure data exchange between health care providers in the two sectors [19]. Selected health records in the HA’s electronic patient record (ePR) system were shared with other public and private health care organizations upon express consent of the patient. The information included patient identity card number, age, gender, diagnosis, procedures codes, discharge summaries, laboratory and radiology reports, medication orders, allergies, and future appointments. Details on the design and implementation of the PPI-ePR have been published elsewhere [17]. Mass media and social media promotions, campaigns, professional seminars, and financial incentives were adopted to implement the project.

As of March 12, 2016, the program covers all 44 public hospitals, 11 private hospitals, and 72 nongovernmental organizations providing health services in 403 residential centers or institutions. ePRs have been accessed more than 1,462,000 times [20]. However, 46% of enrolled patients and 23% of enrolled health care providers had not made use of the system to access patient records since enrollment [21]. Our study, therefore, aimed to investigate the acceptance, awareness, perception, and satisfaction toward the system, supporting further improvement of the eHR in Hong Kong.

## Methods

### Objectives

This project aimed to conduct surveys on the PPI-ePR with the following objectives: (1) evaluate the awareness, acceptance, and perceived benefits of the PPI-ePR among enrolled users; (2) study factors hindering the participation of private doctors and patients in the PPI-ePR; (3) assess the reasons for not using the system after having enrolled; and (4) collect residents’ suggestions on facilitating public acceptance and use of the PPI-ePR. Our findings would provide direction for the design, development, and operation of the eHR system in Hong Kong.

### Sampling

Regarding patients, we adopted simple random sampling to survey enrollees and nonenrollees of the PPI-ePR. Enrollees were randomly selected based on an enrollment list provided by the HA (n=246,000), while nonenrollees were selected from the Hong Kong telephone directory. Computer-generated numbers were used for subject recruitment. Sample sizes were calculated by the formula n=4×p(1–p)/[precision]^2^, where p=proportion of interests. We assumed 50% as the proportion of interests in all outcomes which would give the maximum sample size since the project is the first electronic system to integrate private and public health records in Hong Kong. The minimum sample size was 400 for enrolled patients, given an assumed precision level of .05. Among patients not previously enrolled in the PPI-ePR, a higher precision level was needed due to the heterogeneity of this patient group. Setting a precision level of .03, the minimum sample size was 1111 for nonenrolled patients.

Regarding doctor subjects, self-administered surveys were sent by postal mail to all 4229 registered private doctors (postal addresses were provided by the HA) in Hong Kong. Response rate of Hong Kong doctors in previous surveys was as low as 15% [17]. To maximize our sample, multiple postal mails were sent to doctors who did not respond in the first round. A total of 10,285 survey invitations were mailed.

### Survey Instruments

Our survey was developed based on previously validated surveys and scales. The following constructs were involved in both patient and doctor surveys:

- Knowledge, attitude, and practice questions of enrolled and nonenrolled subjects. People’s awareness and use level of the information-sharing platform were part of our focus
- Constructs of the health belief model were used to understand enroller and nonenroller behavior. Perceived barriers and benefits of participating or using the PPI-ePR program, self-efficacy among doctors, and cues to registration were the main sections of our questionnaires
- User satisfaction levels and doctor suggestions on improving the online platform were collected to inform the design and promotion of future territory-wide patient information sharing projects
- Sociodemographic information was collected for subgroup analysis

Drafted questionnaires were face-validated by a panel of epidemiologists, doctors, nursing professionals, public health practitioners, and academics and subsequently pilot-tested on 20 doctors and 20 patients. Revisions were made after the pilot testing to promote feasibility and item comprehensiveness. Patient and doctor surveys were available in both Chinese and English versions. Our study is nonanonymous. Participants were informed that all information presented would be at the aggregate level, which could not identify any individuals. Consent was sought verbally for phone interviews and was signed by the participants of postal surveys.

### Statistical Analysis

All surveys were checked for their completeness and the presence of participant consent before data entry and analysis with SPSS Statistics version 18.0 (IBM Corp). As part of quality control, validity, quality, and accuracy were randomly checked for both doctor and patient data. Thereafter, items in both surveys were stratified according to the status of enrollment. Descriptive statistics including proportions, means, and standard deviations were presented for doctor users, doctor nonusers, patient users, and patient nonusers separately.

## Results

### Participant Characteristics

The response rates were 90.3% (501 completed surveys/555 telephone number dialed) and 73.4% (1191/1623) for enrolled and nonenrolled patients, respectively. Nonusers were on average younger than users of the PPI-ePR system. Compared with users, a higher percentage of nonusers had tertiary education (nonusers: 26.8% [322/1200] vs users: 18.4% [92/501]) or were a student (11.8% [59/501] vs 1.6% [19/1200]), housewife (17.4% [87/501] vs 15.0% [180/1200]) or full-time employee (43.3% [217/501] vs 33.2% [398/1200]). The median household incomes per month were HK $10,000 (US $1180) and HK $20,000 (US $2361) for enrolled and nonenrolled respondents, respectively (Multimedia Appendix 1).

A total of 10,285 postal invitations were sent to a list of all 4229 registered private doctors in Hong Kong. We received 610 completed postal surveys consisting of 409 enrolled doctors, 198 nonenrolled doctors, and three with unknown enrollment status. The response rate was 14.4% (610 completed surveys/4229 private doctors in Hong Kong as of August 2012). In general, the majority of enrolled doctors were aged 30 to 50 years, while the majority of nonenrolled doctors were aged 51 years or above, indicating a younger group of users compared with nonusers of the system. In addition, nonenrolled doctors were more likely to have longer practice experience after medical school graduation (51.0% [101/198] having more than 30 years) than enrolled doctors (29.1% [119/409] having more than 30 years). The percentage being in solo practice was higher among nonenrolled doctors (71/109, 65.4%) than enrolled doctors (227/409, 55.5%). More enrolled doctors were academy fellows than nonenrolled doctors (68.2% [135/198] versus 56.6% [56/99]; Multimedia Appendix 1).

### Awareness, Acceptance, and Perceived Benefits and Obstacles of Using the Public-Private Interface–Electronic Patient Record

More than 99% (499/501, 99.6%) of enrolled patients were aware of the operation of the PPI-ePR system compared with only 26.2% (314/1200) among nonenrolled patients. Compared with nonenrolled patients, enrolled patients enjoyed more ways to discover the project, among which family doctor’s recommendation (305/501, 60.9%) and seminars organized by the HA (85/501, 17.0%) were widely praised; yet for nonenrolled patients, TV and newspapers dominated their understanding toward the system. The sharp disparity of awareness between users and nonusers was not observed among doctors—74.7% (148/198) of the surveyed doctors were aware of the project although they had not enrolled. Peers in the health care sector and posters or leaflets from the HA were the most effective means to promote PPI-ePR among both enrolled and nonenrolled doctors.

To further understand subject enrollment and use of PPI-ePR, we asked enrolled patients and doctors about their perceived benefits of joining the system and asked nonenrolled patients and doctors about factors hindering their enrollment.

Improved health care quality, convenience, and economic incentives were among the benefits of the PPI-ePR system emphasized by enrolled patients (Table 1). Specifically, about half of project users perceived reduced repetition of health assessment and information provision (266/501, 53.1%), as well as easier access to doctors’ recommendations (217/501, 43.3%). Improved health care quality was the most frequently reported benefit of the PPI-ePR system among enrolled doctors, followed by improved patient safety. Specifically, the system facilitated timely access to patient medical records (314/409, 76.8%), enhanced continuity of patient care (259/409, 63.3%), and smoothed delivery of health care services (249/409, 60.9%).

**Table 1:** Perceived benefits of the Public-Private Interface–Electronic Patient Record among users.

Perceived benefits among users			Persons, n (%)
**Enrolled patients (n=501)**			
	**Improved health care**		
		Reduce duplicated tests and treatments	266 (53.1)
		Access doctor recommendation	217 (43.3)
		Comprehensive medical records for better patient care	144 (28.7)
		Fluent information flow between private and public health care sectors	100 (20.0)
		Facilitate continuity of patient care	5 (1.0)
	**Convenience**		
		No need to bring medical reports	48 (9.6)
	**Economic incentive**		
		Souvenirs	7 (1.4)
**Enrolled doctors (n=409)**			
	**Improved health care quality**		
		Timely access to patient medical records in the Hospital Authority	314 (76.8)
		Facilitate continuity of patient care	259 (63.3)
		Delivery of health care service	249 (60.9)
	**Safety**		
		Improved patient safety	209 (51.1)

Lack of awareness was the most common obstacle preventing nonenrolled patients (483/1200, 40.3%) from joining the system (Table 2), followed by not being clear about project objectives (229/1200, 19.1%) and high levels of self-perceived health status (157/1200, 13.1%). Among nonenrolled doctors, feasibility and benefits of the system were widely challenged; 48.0% (95/198) of nonenrolled doctors complained about the complicated enrollment procedure and 40.4% (80/198) were hindered by the additional workload of migrating data from paper records to computers.

**Table 2:** Top five hindering factors for enrolling in the Public-Private Interface–Electronic Patient Record system.

Perceived barriers among nonusers			Persons, n (%)	
**Nonenrolled patients (n=1200)**				
	**Low awareness**			
		Unaware of the project	483 (40.3)	
		Unclear about project objectives	229 (19.1)	
	**Low necessity**			
		High levels of self-perceived health	157 (13.1)	
	**Safety reasons**			
		Concern about safety of personal data and privacy	150 (12.5)	
	**Time-consuming**			
		I don’t have enough time	93 (7.8)	
**Nonenrolled doctors (n=198)**				
	**Low feasibility**			
		Complicated procedure to join the project	95 (48.0)	
		Concerns about additional workload for data migration from paper records to computer	80 (40.4)	
		Viewing electronic medical records is time-consuming	60 (30.3)	
	**Unclear benefits**			
		Use of the system does not assist clinical operation and provide significant benefits	58 (29.3)	

A Hong Kong Government report showed that a large proportion of subjects had not made use of the system to access patient records since enrollment [20]. Hence, our study explored the reasons people registered but did not use the system. A total of 44.5% (223/501) of surveyed patients had not used the system after enrollment as they did not have a clinical indication requiring PPI-ePR access (153/223, 68.6%), did not know how the system works (22/223, 9.9%), and/or their doctor had not enrolled (22/223, 9.9%; Table 3). Among the 409 enrolled doctors, 22 (5.4%) had never accessed their patients’ medical records via the PPI-ePR. For 22 doctors who had not accessed the PPI-ePR after joining and gave a reason, the most commonly reported reason was the absence of clinical indication requiring PPI-ePR access (8/21, 38.1%) and forgotten log-in password (5/21, 23.8%; Table 3).

**Table 3:** Reasons for not using the Public-Private Interface–Electronic Patient Record system after enrollment.

Reasons for not using system after enrolling		Persons, n (%)
**Enrolled patients (n=223)**		
	No clinical indication of need for accessing PPI-ePR^a^ data	153 (68.6)
	Don’t know how to use	22 (9.9)
	My family doctor does not participate	22 (9.9)
	Password forgotten	16 (7.2)
**Enrolled doctors (n=22)**		
	No clinical indication of need for accessing PPI-ePR data	8 (38.1)
	Password forgotten	5 (23.8)
	Patient has not joined PPI-ePR project	4 (19.0)
	Patient failed or refused to provide log-in details to authorize the access	4 (19.0)
	Cannot afford time to trace the records	1 (4.8)

^a^PPI-ePR: Public-Private Interface–Electronic Patient Record.

### User Satisfaction

Enrolled patients, in general, had higher satisfaction levels than enrolled doctors (*P* < 0.001). Around 10% ([40+10]/409, 12.2%) of enrolled doctors were dissatisfied or very dissatisfied with the PPI-ePR online system, which is far higher than that among enrolled patients ([1+1]/501, 0.4%; Table 4).

**Table 4:** Users' satisfaction levels with the Public-Private Interface–Electronic Patient Record online system.

Level of satisfaction	Doctors (N=409), n (%)	Patients (n=501), n (%)
Very satisfied	40 (9.8%)	18 (16.5%)
Satisfied	238 (58.1%)	300 (59.9%)
Neutral	81 (19.9%)	116 (23.2%)
Dissatisfied	40 (9.8%)	1 (0.2%)
Very dissatisfied	10 (2.4%)	1 (0.2%)

### Suggested Cues to Registration

Our survey also asked patients and doctors for suggestions on increasing the PPI-ePR registration rate. Television, newspaper, and magazine advertisements were regarded as the most effective means to enhance public knowledge and encourage participation in the program among the users and nonusers (1297/1701, 76.2%), followed by recommendation from health care professionals (248/1701, 14.6%; Table 5). Among doctors, medical profession newsletters or journals (428/610, 70.2%) were reported as the most popular promotional strategies. Onsite promotional activities (217/610, 35.6%); television, newspaper, and magazine advertisements; and websites (183/610, 30.0%) were also commonly suggested.

**Table 5:** Suggested cues to promote the registration of Public-Private Interface–Electronic Patient Record.

Suggested cues by user group		Persons, n (%)	
**Patients (n=1701)**			
	Television, newspaper, and magazine advertisements		1297 (76.2)
	Recommendations from health care professionals		248 (14.6)
	Posters/leaflets		245 (14.4)
	Organized seminars		118 (6.9)
	Governmental and Hospital Authority websites		78 (4.6)
	Souvenirs		5 (0.3)
**Doctors (n=610)**			
	Medical profession newsletters and journals		428 (70.2)
	Onsite promotion activities		217 (35.6)
	Television, newspaper, and magazine advertisements		183 (30.0)
	Website		172 (28.2)
	Posters/leaflets		104 (17.0)
	Social media (eg, Facebook)		66 (10.8)
	Incentives and souvenirs		61 (10.0)

### Doctor Suggestions on Improving Public-Private Interface–Electronic Patient Record Online Platform

The extension of sharable data scope (224/409 enrolled doctors agreed, 54.8%) has been frequently reported as an area for future improvement among enrolled doctors, followed by a more user-friendly interface (61/198, 30.8%). Nonusers of the system believed simplification of the enrollment process (107/198, 54.0%) and technical support for operations (90/198, 45.5%) would be the most effective ways to improve the project (Table 6).

**Table 6:** Doctor suggestions for improving the Public-Private Interface–Electronic Patient Record Project system.

Suggestions for improving the online platform	Enrolled doctors (n=409), n (%)	Nonenrolled doctors (n=198), n (%)
Simplification of enrollment process	91 (22.2)	107 (54.0)
Technical support for system operation	54 (13.2)	90 (45.5)
Develop user-friendly interface	126 (30.8)	61 (30.8)
Extend sharable data scope	224 (54.8)	11 (5.6)

## Discussion

### Principal Findings

Territory-wide, large degree, timely, legal, and secure health information exchange (HIE) is necessary to improve health care quality, especially in Hong Kong where the private and public health care sectors are autonomous and independent units with little or no experience in information sharing. This study is the first to provide a comprehensive assessment on the awareness, perceptions, obstacles, and suggestions for registering and using the PPI-ePR online interface among both patients and doctors.

We revealed a much lower awareness level of HIE in Hong Kong than in other Asia-Pacific regions emphasizing computerized information exchange among hospitals [22]. Countries like Australia, Malaysia, the United Kingdom, and the United States had widely adopted, existing infrastructures of cloud computing and/or basic eHR platforms before the implementation of the territory-wide platform [18]. In Hong Kong, however, a large proportion of doctors were deeply involved in scattered, paper-based health records before joining the PPI-ePR project. The implementation of health information technology hence started from the idea germination stage, requiring an even longer time and more multidisciplinary promotions [23]. Mass media may be the most effective strategy to increase public awareness, according to our survey results. Western experience, however, emphasized the power of significant others, including family doctors and peers, in motivating patient enrollment [24]. Literature on health behaviors discussed both types of promotions. It has been summarized that public promotions were more effective in increasing individual awareness, especially in the earlier stages of innovation implementation [25]. Among populations with high levels of awareness and acceptance of the innovation, strong ties and small-group interactions were more essential for behavior modification [22]. Our survey results echoed this idea. Although both ways were praised by enrolled and nonenrolled patients, enrolled patients reported family and doctor recommendations as the most popular way of knowing about PPI-ePR, while nonenrolled patients rated television and newspaper as the most popular ways.

Regarding perceived barriers, although concerns about privacy and data security have been widely discussed in global literature [26,27], few patients and doctors in Hong Kong were worried about data confidentiality issues. Scholars in Bangladesh, where the dual-track health care system is very similar to Hong Kong, also found no association between data privacy and the use of eHealth systems [28]. Patients’ low bargaining power in the policy decision process and the absence of health privacy awareness were suggested as reasons for the low concerns in data confidentiality. Another qualitative study in the United States suggested that patient concerns about privacy and data security were mostly nonspecific, gut-level emotions that may be properly soothed by public education [29]. The low level of worries in Hong Kong may benefit from the repeated stress of encrypted and safeguard algorithms and constant emphasis on patients’ legal rights in most promotional materials. All functions and powers of PPI-ePR are required under the Electronic Health Record Sharing System Ordinance (Chapter 625), with a well-developed complaint handling policy [30].

Our results echoed previous literature [31,32] reporting reducing duplicate tests and treatments and timely access to patients’ medical records as the main benefits of HIE projects like PPI-ePR. Despite the perceived benefits, we noted low use levels of the information system, which has also been observed in studies in Australia, Europe, and some states in the United States [31,33]. Such low use levels hinder the effectiveness of building a health information system. A recent systematic review of 22 population-based studies summarized that regardless of the compatibility and efficacy of EHR systems, emergency department patients with severe symptoms, elderly patients with chronic disease, and those who had a recent admission history were more likely to have encounter-related use [34]. Future development and implementation of HIE projects in Hong Kong may consider more on these populations.

Our study also revealed that patient and doctor levels of use were dependent on each other. Nonenrollment of doctors is a popular reason for patients to not make use of the PPI-ePR platform. A lack of clinical indication required data extraction from other hospitals was among the main reasons for the low level of PPI-ePR use. The current PPI-ePR project only allows the exchange of limited types of data and does not allow patients access to their own records. Studies in the United Kingdom, United States, and Germany emphasized multiple functions of EHR which may encourage active use. These functions included recommendations for daily exercise, prescription refill, outpatient reservation, and appointment reminders [33,35,36]. Their experience may be adapted to Hong Kong to attract patient and doctor use. The HA in Hong Kong may consider enlisting enrolled doctors to clarify the simplicity of the enrollment procedure, as peers in the health care sectors were shown to be common means of learning about the PPI-ePR.

Among enrolled users of the PPI-ePR, over 76% of patients and 67% of doctors were satisfied with its overall performance. A user-friendly interface, simple enrollment procedures, good training, technical information support, and smooth transition with little migration efforts were regarded as important for improving doctors’ satisfaction. Scholars from Malaysia also noted these needs especially among elder doctors who lacked computer skills, even 30 years after the EHR implementation [37], indicating the continuous needs of training and supports. Meanwhile, the dilemma between simplified data exchange procedure and data confidentiality was discussed in global studies. The current PPI-ePR system adopted a 2-factor authentication process to safeguard patient privacy. Each access to patient health records required passwords from both patients and authorized doctors, while both static password and a dynamic password generated from the security token were required on the doctors’ side. Such a procedure has been found to be satisfactory following both external and internal audits [20] but required extra time and efforts from care givers. Nevertheless, ongoing modification of the online portal based on feedback from medical staff is the key to increasing use and satisfaction levels among doctors [38]. It is noted that HA and the Electronic Health Record Registration Office in Hong Kong have been considering doctors’ suggestions and formulating technical details and logistic arrangements for a more effective migration plan.

### Strengths and Limitations

This study is the first to comprehensively assess the acceptance and practice of the EHR system in Hong Kong. Although the benefits, facilitators, and barriers of EHRs have been widely discussed in countries including the United States [38], United Kingdom [33], Germany [39], and Australia [40] in the past decade, much effort is needed in the Eastern world, where EHRs have generally been established only in the past few years [40,41]. Previous assessments mainly focused on either the patient or doctor side [8,10,42,43]. Our research involved both sides, comparing the perceptions among enrolled patients, nonenrolled patients, enrolled doctors, and nonenrolled doctors. Results provided comprehensive evaluations of the first EHR project interface between the public and private health sectors in Hong Kong.

This study has some limitations. Our survey combined widely used questions on knowledge, attitudes, and practices studies and health belief model studies [44,45]. Some questions were rephrased to ensure their fitness in Hong Kong and have only undergone face validity by our expert panel. Results of this localized survey may only be adapted to Hong Kong society and may not be generalizable. For doctors’ surveys, the sampling frame included all private medical doctors in Hong Kong from a comprehensive list of postal invitations. The response rate was, nevertheless, only 14.4%, with a large difference between enrolled and nonenrolled doctors. Until 2016, fewer than 1930 private doctors had enrolled in the PPI-ePR system. Our sample covered over 21.1% of the enrolled population. Yet for nonenrolled doctors, the coverage rate was less than 10%, introducing a form of response bias later studies may pay attention to.

### Implication

The PPI-ePR project provided a backbone for the territory-wide Electronic Health Record Sharing System (eHRSS), which was launched in March 2016 [46]. Since the development of eHRSS was heavily influenced by the PPI-ePR, further studies may compare the acceptance, barriers, facilitators, and satisfaction among various stakeholders between the PPI-ePR and eHRSS systems and assess trends of eHR adaptation in Hong Kong.

### Conclusions

This study explored the attitudes, practices, barriers, and facilitators of joining PPI-ePR in Hong Kong. Findings were consistent with some global challenges of promoting territory-wide eHRs while specific features in Hong Kong were also noted. For one, comparing the high levels of satisfaction among users and limited awareness among nonusers, efforts should be paid at this stage on the behavioral change of nonusers. On the other hand, since the PPI-ePR is migrating toward the eHRSS, continuous engagement of existing users is equally important. The benefits of sharing health records between the private and public health care sectors, as well as that between patients and doctors, should be further promoted through medical journals, newspapers, and television advertisements. Meanwhile, the information technology system should be more user-friendly with more functions involved to encourage continuous use after enrollment. More promotion and simplified information technology operations can help to ensure the acceptance and effectiveness of eHR sharing in Hong Kong.

## Appendix

Multimedia Appendix 1 Patient and doctor characteristics.

## Abbreviations

EHR: electronic health record
eHRSS: Electronic Health Record Sharing System
EMR: electronic medical record
ePR: electronic patient record
HA: Hospital Authority
HIE: health information exchange
PPI-ePR: Public-Private Interface–Electronic Patient Record
