# Supplementary material for: Adoption of an Electronic Patient Record Sharing Pilot Project: Cross-Sectional Survey
Source: J Med Internet Res. 2020 Apr 6;22(4):e13761. doi: 10.2196/13761 (PMC7171565; doi:10.2196/13761)
Supplement: Multimedia Appendix 1 [file jmir_v22i4e13761_app1.docx]

| Table 1a. Basic Characteristics of Patients | | | | | | | |
| --- | --- | --- | --- | --- | --- | --- | --- |
|  | Patient (N=1,701) | | | | | |  |
|  | Total  (N=1,701) | | Enrolled (N=501) | | Non-enrolled (N=1,200) | |  |
|  | N | % | N | % | N | % | p |
| **Sex** |  |  |  |  |  |  | .20 |
| Male | 775 | 45.6 | 216 | 43.1 | 559 | 46.6 |  |
| Female | 925 | 54.4 | 284 | 56.7 | 641 | 53.4 |  |
| Refused to answer | 1 | 0.1 | 1 | 0.2 | - | - |  |
| **Age, years** | |  |  |  |  |  | < .001 |
| 16-25 | 184 | 10.8 | 11 | 2.2 | 173 | 14.4 |  |
| 26-35 | 230 | 13.5 | 26 | 5.2 | 204 | 17.0 |  |
| 36-45 | 272 | 16.0 | 52 | 10.4 | 220 | 18.3 |  |
| 46-55 | 353 | 20.8 | 120 | 24.0 | 233 | 19.4 |  |
| 55-65 | 306 | 18.0 | 130 | 25.9 | 176 | 14.7 |  |
| >65 | 356 | 20.9 | 162 | 32.3 | 194 | 16.2 |  |
| **Educational level** | |  |  |  |  |  | < .001 |
| Illiterate/Kindergarten/Primary | 424 | 24.9 | 158 | 31.5 | 266 | 22.2 |  |
| Secondary | 858 | 50.4 | 250 | 49.9 | 608 | 50.7 |  |
| Tertiary or above | 414 | 24.3 | 92 | 18.4 | 322 | 26.8 |  |
| Refused to answer | 5 | 0.3 | 1 | 0.2 | 4 | 0.3 |  |
| **Employment Status** | |  |  |  |  |  | < .001 |
| Unemployed | 50 | 2.9 | 20 | 4.0 | 30 | 2.5 |  |
| Employed | 749 | 44.0 | 184 | 36.7 | 565 | 47.1 |  |
| Student/housewife | 432 | 25.4 | 83 | 16.6 | 349 | 29.1 |  |
| Retired | 462 | 27.2 | 213 | 42.5 | 249 | 20.8 |  |
| Refused to answer | 8 | 0.5 | 1 | 0.2 | 7 | 0.6 |  |
| **Monthly household income (HK$)** | | | |  |  |  | .02 |
| 0 – 10,000 | 474 | 27.9 | 156 | 31.1 | 318 | 26.5 |  |
| 10,001 – 20,000 | 359 | 21.1 | 99 | 19.8 | 260 | 21.7 |  |
| 20,001 – 30,000 | 254 | 14.9 | 64 | 12.8 | 190 | 15.8 |  |
| 30,001 – 40,000 | 132 | 7.8 | 26 | 5.2 | 106 | 8.8 |  |
| >40,000 | 237 | 13.9 | 74 | 14.8 | 163 | 13.6 |  |
| Refused to answer | 245 | 14.4 | 82 | 16.4 | 163 | 13.6 |  |

| Table 1b. Basic Characteristics of Doctors | | | | |  |  |  |
| --- | --- | --- | --- | --- | --- | --- | --- |
|  | Doctors (N=607)* | | | | | |  |
|  | Total  (N=607) | | Enrolled (N=409) | | Non-enrolled (N=198) | |  |
|  | N | % | N | % | N | % | p |
| **Sex** |  |  |  |  |  |  | < .001 |
| Male | 484 | 79.7 | 340 | 83.1 | 144 | 72.7 |  |
| Female | 123 | 20.3 | 69 | 16.9 | 54 | 27.3 |  |
| **Age, years** | |  |  |  |  |  | < .001 |
| <30 | 2 | 0.3 | 1 | 0.2 | 1 | 0.5 |  |
| 31-40 | 94 | 15.5 | 68 | 16.6 | 26 | 13.1 |  |
| 41-50 | 177 | 29.2 | 135 | 33.0 | 42 | 21.2 |  |
| 51-60 | 176 | 29.0 | 121 | 29.5 | 55 | 27.8 |  |
| >60 | 158 | 26.0 | 84 | 20.6 | 74 | 37.4 |  |
| **Year of practice, Years** | | |  |  |  |  | < .001 |
| 0-15 | 113 | 18.6 | 82 | 20.0 | 31 | 15.7 |  |
| 16-30 | 274 | 45.1 | 208 | 50.9 | 66 | 33.3 |  |
| >30 | 220 | 36.2 | 119 | 29.1 | 101 | 51.0 |  |
| **Type of Clinical Practice** | | |  |  |  |  | < .001 |
| Solo Practice | 298 | 49.1 | 227 | 55.5 | 71 | 35.9 |  |
| With partners/Group practice | 146 | 24.1 | 117 | 28.6 | 29 | 14.6 |  |
| Private Hospital | 61 | 10.0 | 57 | 13.9 | 4 | 2.0 |  |
| Others | 13 | 2.1 | 8 | 2.0 | 5 | 2.5 |  |
| Missing | 89 | 14.7 | - | - | 89 | 44.9 |  |

* Three doctors did not remember whether they enrolled the PPI-ePR or not.
